# Dual‐Lineage Chondrocyte‐Like Cells in the Nucleus Pulposus of Aging Intervertebral Discs Are Accelerated by Hedgehog Signaling Inactivation

**DOI:** 10.1111/acel.70248

**Published:** 2025-09-22

**Authors:** Lei Zhang, Chunmei Xiu, Hongji You, Jianquan Chen

**Affiliations:** ^1^ Orthopedic Institute Suzhou Medical College, Soochow University Suzhou Jiangsu China; ^2^ School of Medicine Hangzhou City University Zhejiang Hangzhou China

**Keywords:** chondrocyte‐like cells, hedgehog signaling, intervertebral disc degeneration, lineage tracing, nucleus pulposus

## Abstract

Intervertebral disc (IVD) degeneration, a major contributor to chronic low back pain, is characterized by the age‐related replacement of notochord‐derived nucleus pulposus cells (NPCs) with chondrocyte‐like or fibrotic cells (CLCs). However, the cellular origins of CLCs and mechanisms driving their emergence remain contentious. Using genetic lineage tracing with *Shh‐Cre* and *Gli1‐CreER* ^*T2*^ to track notochordal and non‐notochordal cells, respectively, we demonstrate that CLCs arise from dual lineages: notochordal NPCs and non‐notochordal Gli1^+^ progenitors. We identified three CLC subtypes, including nested (N‐CLCs), clustered (C‐CLCs), and disordered (D‐CLCs), with distinct morphological and/or molecular profiles. N‐CLCs and C‐CLCs originate from NPCs, whereas D‐CLCs derive from Gli1^+^ cells infiltrating the NP. Furthermore, conditional ablation of *Smo*, an essential transducer of Hh signaling, in adult discs accelerated degeneration and promoted both NP‐derived (Krt19^+^ N‐CLCs) and non‐NPC‐derived (Krt19^−^ D‐CLCs) populations. These results establish that Hh signaling suppresses dual‐lineage CLC expansion during aging. Our findings resolve controversies surrounding CLC origins, delineate their dynamic progression during degeneration, and highlight Hh signaling as a promising therapeutic target to counteract pathological cell fate shifts in aging discs.

## Graphical abstract

During intervertebral disc degeneration, notochord‐derived nucleus pulposus cells progressively transdifferentiate into Krt19^+^ chondrocyte‐like cells (CLCs) and Krt19^−^ CLCs, which are eventually replaced by Krt19^−^ CLCs from non‐notochordal Gli1^+^ progenitors. Inactivation of Hedgehog signaling promotes the expansion of both notochordal and non‐notochordal CLC populations in mice.

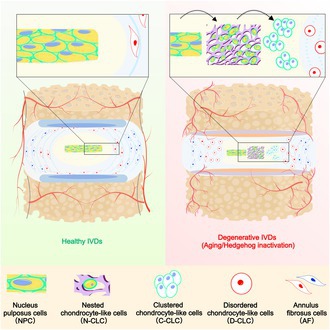

1

Intervertebral disc (IVD) degeneration, a primary cause of debilitating low back pain, results from the structural breakdown of IVDs, which are composed of the gelatinous and proteoglycan‐rich nucleus pulposus (NP), collagenous annulus fibrosus (AF), and cartilaginous endplates (CEP) (Knezevic et al. 2021; Novais et al. 2020; Zhang et al. 2024). Aging is the dominant driver of this process, marked by the NP's transition from notochordal to fibrocartilaginous tissue in both humans and mice (Au et al. 2020; Mohanty et al. 2019; Novais et al. 2020; Roberts et al. 2006; Weiler et al. 2010; Yang et al. 2009). This shift reduces the NP's compressive resilience, leading to disc degeneration or herniation. Cellularly, large vacuolated NP cells are progressively replaced by chondrocyte‐like or fibroblastic cells (collectively termed as CLCs hereafter) residing within a dense pericellular or extracellular matrix (Au et al. 2020; Mohanty et al. 2019; Roberts et al. 2006; Weiler et al. 2010; Winkler et al. 2014; Yang et al. 2009). Notably, these degenerative alterations are also recapitulated in mouse models of injury‐induced IVD degeneration (Yang et al. 2009).

While NP cells (NPCs) originate from embryonic notochord (Choi et al. 2008; McCann et al. 2012; Zheng et al. 2019), the source of CLCs remains contested. Lineage tracing using *Krt19‐CreER* suggests that CLCs are transdifferentiated NPCs (Au et al. 2020; Mohanty et al. 2019). However, conflicting studies propose non‐notochordal origins (Kim et al. 2003). These discrepancies may reflect differences in cellular sources at distinct stages of degeneration. Consistent with this concept, Au et al. showed that during injury‐induced disc degeneration in mice, both NPC‐derived and non‐NPC‐derived CLCs populate fibrotic discs (Au et al. 2020). Beyond their cellular origin, the mechanisms driving the age‐related appearance of CLCs are also poorly defined. Addressing these questions is critical for developing targeted therapies to prevent or treat disc degeneration. In this study, we employed multiple genetic mouse models to determine the contribution of both NPCs and non‐NPC lineage cells to CLCs in the aging discs, and to elucidate the role of Hedgehog (Hh) signaling in their formation.

To investigate whether NPCs directly transdifferentiate into CLCs during aging, we utilized *Shh‐Cre; R26‐mT/mG* mice, in which notochordal lineage cells (including NPCs and their descendants) are labeled with EGFP (mG), while non‐notochordal cells express Tomato (mT). IVDs from 16‐ to 24‐month‐old mice were analyzed via histology and immunohistochemistry for Krt19, an NPC marker. We observed significant heterogeneity in the NP cell population. In addition to typical reticular NPCs, which express Krt19 and organize into either a continuous or partially dispersed band (Tam et al. 2018), we identified three distinct subtypes of CLCs: (1) Nested CLCs (N‐CLCs) segregated by dense extracellular matrix (ECM) with most expressing Krt19; (2) Clustered CLCs (C‐CLCs), forming larger multicellular aggregates (> 3 cells) that are largely Krt19^−^; and (3) Disordered CLCs (D‐CLCs), which appeared as Krt19^−^ single cells or small clusters (2–3 cells) lacking a defined structural arrangement (Figure S1). Consistent with their chondrocyte‐like identity, the majority of CLCs across all subtypes either strongly expressed collagen X (ColX), a hypertrophic chondrocyte marker, or were enveloped by ColX‐rich pericellular or extracellular matrix (Figure S2). More importantly, nearly all NPCs showed mG fluorescence and Krt19 expression. N‐ and C‐CLCs were also mG‐labeled, but most C‐CLCs lost Krt19 (Figure 1a–e). In contrast, the vast majority of D‐CLCs lacked mG and Krt19 signals (Figure 1a–e). Similar cell types were found in aging human discs (Weiler et al. 2010). Notably, the co‐existence of C‐CLCs and D‐CLCs was also observed in certain IVDs (Figure S3). Quantitatively, all aging mice (*n* = 6) exhibited a mix of NPC‐dominant and CLC‐dominant discs, with no strict age correlation. Of 42 IVDs analyzed, 59.5% were entirely NPCs, 11.9% had N‐CLCs, and the rest were predominantly C‐ or D‐CLCs (Figure 1f). These categories showed significant histological score differences, as assessed using a validated grading system (Tam et al. 2018) (Figure 1g). These results indicate that N‐CLCs and C‐CLCs originate from notochordal cells, representing distinct transitional stages, whereas D‐CLCs likely arise from non‐notochordal cells infiltrating the NP region.

**FIGURE 1:**
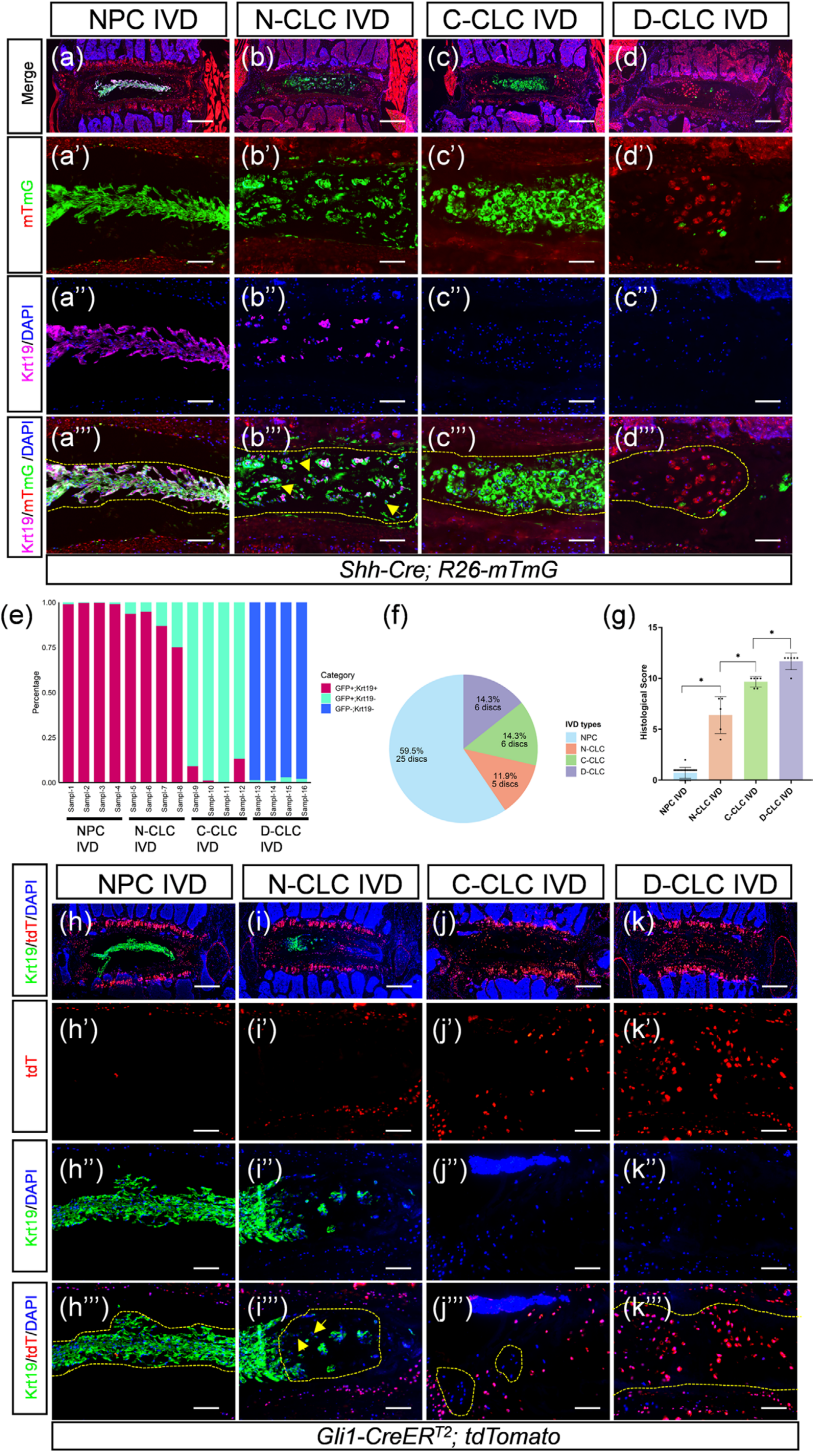
Chondrocyte‐like cells in the nucleus pulposus of aging intervertebral discs are derived from both notochordal and non‐notochordal cells lineages. (a–d′′′) Fate‐mapping of notochordal cells in the NPC, N‐CLC, C‐CLC, or D‐CLC IVD of 16‐ to 24‐month‐old *Shh‐Cre; mT/mG* mice. (a–d) Low‐power combined visualization of mT (red), mG (green), DAPI (blue), and Krt19 (magenta) immunofluorescence across entire IVDs. High‐power views of the NP region are shown in (a′–a′′′), (b′–b′′′), (c′–c′′′), and (d′–d′′′), respectively. (a′–d′) mT (red) and mG (green) epifluorescence. (a′′–d′′) DAPI (blue) and Krt19 (magenta) immunofluorescence. (a′′′–d′′′) Combined visualization of mT (red), mG (green), DAPI (blue), and Krt19 (magenta). Encircled NP regions in (a′′′–d′′′) indicate nucleus pulposus cells (NPCs), nested chondrocyte‐like cells (N‐CLCs), clustered chondrocyte‐like cells (C‐CLCs), and disordered chondrocyte‐like cells (D‐CLCs), respectively. Yellow arrows in (b′′′) highlight N‐CLCs lacking Krt19 expression. (e) Quantification of cell subpopulations in the nucleus pulposus (NP) region of intervertebral discs (IVDs) from 16‐ to 24‐month‐old *Shh‐Cre; R26‐mT/mG* mice based on immunofluorescence detection of GFP and Krt19 co‐expression. The percentage of GFP^+^Krt9^+^, GFP^+^Krt19^−^, GFP^−^Krt19^+^, and GFP^−^Krt19^−^ cells was determined in NP regions of discs classified as nucleus pulposus cell‐dominated (NPC), nested chondrocyte‐like cell (N‐CLC), clustered chondrocyte‐like cell (C‐CLC), or disordered chondrocyte‐like cell (D‐CLC) IVDs. Four IVD samples were analyzed per category. (f) Proportion of discs classified as NPC, N‐CLC, C‐CLC, or D‐CLC IVDs among 42 T13‐S1 discs from six 16‐ to 24‐month‐old *Shh‐Cre; mT/mG* mice. NPC IVD: Discs composed entirely of nucleus pulposus cells (NPCs); N‐CLC IVD: Discs containing nested chondrocyte‐like cells; C‐CLC IVD: Discs predominantly occupied by clustered chondrocyte‐like cells; D‐CLC IVD: Discs predominantly occupied by disordered chondrocyte‐like cells. (g) Quantitative analysis of histological scores for NPC, N‐CLC, C‐CLC, and D‐CLC IVDs. Data are presented as mean ± SD, with each dot representing one disc's histological score. Statistical significance was determined using one‐way ANOVA with Tukey's multiple comparisons test. *p < 0.05. (h–k′′′) Lineage‐tracing of non‐notochordal cells in the NPC, N‐CLC, C‐CLC, or D‐CLC IVDs of 16‐ and 19‐month‐old *Gli1‐CreER^T2^; tdTomato* mice. (h–k) Low‐power combined visualization of tdTomato (tdT, red), DAPI (blue), and Krt19 (green) immunofluorescence across entire IVDs. High‐power views of the NP region are shown in (h′–h′′′), (i–i′′′), (j′–j′′′), and (k′–k′′′), respectively. (h′–k′) tdT (red) epifluorescence. (h′′–k′′) DAPI (blue) and Krt19 (green) immunofluorescence. (h′′′–k′′′) Combined visualization of tdT (red), DAPI (blue), and Krt19 (green). Encircled NP regions in (h′′′–k′′′) indicate NPCs, N‐CLCs, C‐CLCs, and D‐CLCs, respectively. Yellow arrows in (i′′′) highlight N‐CLCs lacking Krt19 expression. Scale bar in panels a, b, c, d, h, i, j, k: 400 μm; 100 μm in other panels.

To further validate the contribution of non‐notochordal cells to CLCs in aging NP, we generated *Gli1‐CreER* ^*T2*^; *R26‐tdTomato* mice (Ahn and Joyner 2004; Madisen et al. 2010). In this model, tamoxifen administration at 2 months of age selectively labels non‐NP Gli1^+^ cells with tdTomato (tdT), sparing notochord‐derived NPCs. We obtained two aging mice, both exhibiting discs with NP regions predominantly populated by NPCs or CLCs (Figure S4). These CLCs primarily included N‐CLCs and D‐CLCs (Figure S4). Most CLCs exhibited high levels of ColX expression (Figure S5). Lineage tracing was performed using tdT expression (Figure 1h–k'''). Nearly all NPCs and N‐CLCs, regardless of Krt19 expression, were devoid of tdT labeling. Similarly, C‐CLCs remained tdT‐negative. In contrast, the vast majority of D‐CLCs exhibited tdT fluorescence. These findings further support the conclusion that N‐CLCs and C‐CLCs are of notochordal origin, whereas D‐CLCs derive from non‐notochordal progenitors, specifically Gli1^+^ cells. Contrary to our findings, Mohanty et al. previously proposed that CLCs in aged IVDs are transdifferentiated NPCs that still express Krt19 (Mohanty et al. 2019). However, their conclusion was based on only two CLC‐resident discs, which may did not have Krt19^−^ C‐CLCs and D‐CLCs, since these two CLC subtypes most likely form at later stages of disc degeneration.

Previous studies have suggested that age‐related declines in Hh signaling contribute to CLC accumulation in the aging NP (Dahia et al. 2009; Mohanty et al. 2019; Winkler et al. 2014; Zhang et al. 2024). To directly test this hypothesis, we genetically ablated *Smo*, a key Hh signal transducer, in adult disc cells of tamoxifen‐inducible *Agc1‐CreER* ^*T2*^;*Smo^c/c^* mice (Henry et al. 2009). Unlike juvenile *Smo* deletion, which severely compromises IVD structure (Zhang et al. 2024), *Smo* ablation at 2 months only induced mild IVD degeneration without triggering CLC formation by 6 months of age (Figure S6). Strikingly, by 14 months, *Smo*‐deficient mice exhibited significantly accelerated disc degeneration associated with increased CLC production in the NP (Figure 2). These CLCs mainly consisted of Krt19^+^ N‐CLCs and Krt19^−^ D‐CLCs. Thus, Hh signaling indirectly suppresses dual‐lineage CLC formation during aging.

**FIGURE 2:**
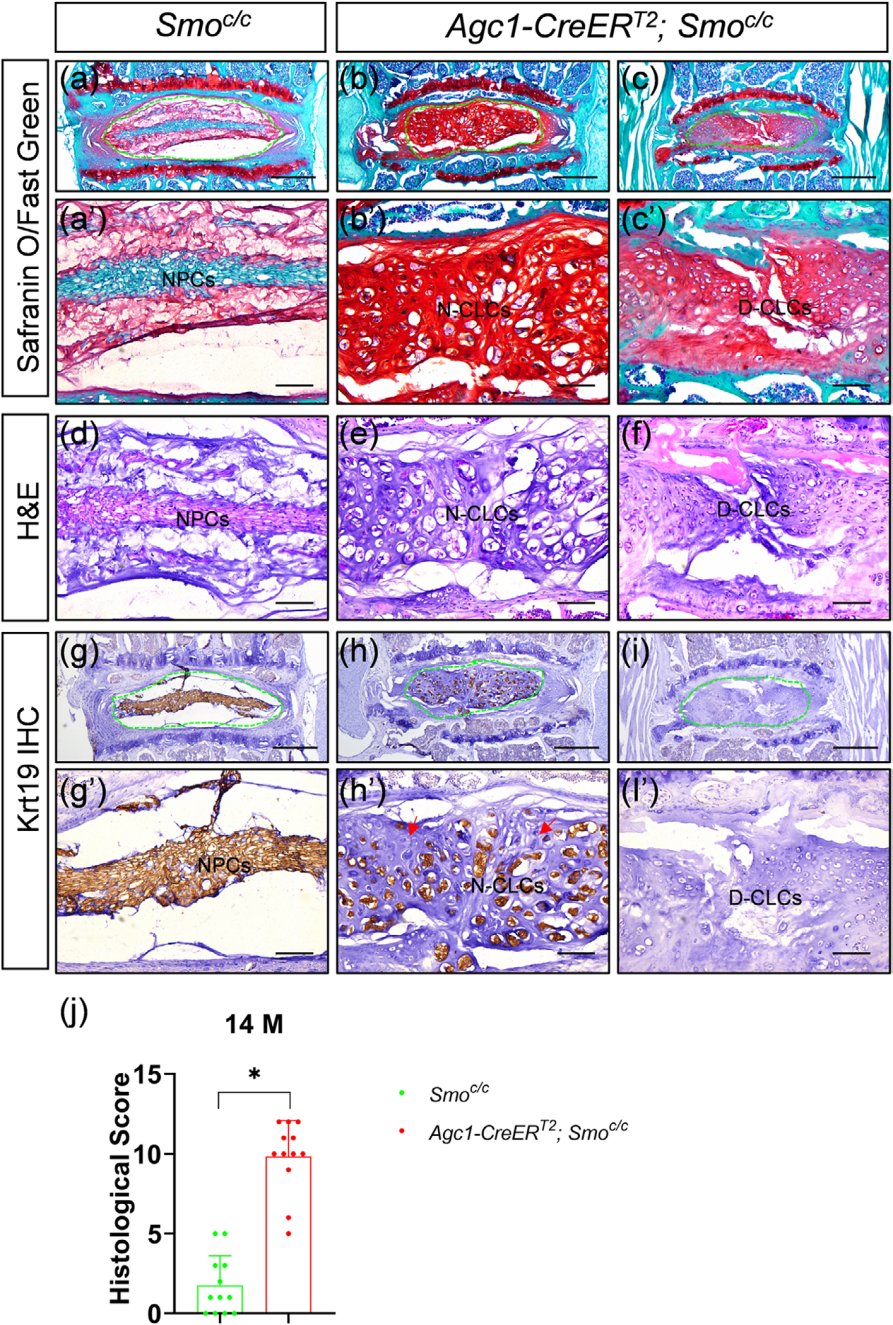
Inactivation of Hedgehog signaling by conditional ablation of *Smo* in intervertebral discs accelerates the appearance of dual‐lineage chondrocyte‐like cells in the nucleus pulposus during aging. (a–f) Safranin O/Fast Green (a–c′), hematoxylin and eosin (H&E) (d–f), and Krt19 immunohistochemical staining (g–i′) of the coronal sections of intervertebral discs from 14‐month‐old *Smo* ^*c/c*^ (a, a′, d, g, g′) and *Agc1‐CreER* ^*T2*^; *Smo* ^*c/c*^ mice (b–c′, e, f, h–i′). Panels a, b, c, g, h, and i show low‐power views of entire intervertebral discs, with the nucleus pulposus region encircled by a green dashed line. Other panels are high‐power views of the nucleus pulposus. Red arrows in (h′) indicate nested chondrocyte‐like cells lacking Krt19 expression. IHC: Immunohistochemistry; NPCs: Nucleus pulposus cells; N‐CLCs: Nested chondrocyte‐like cells; D‐CLCs: Disordered chondrocyte‐like cells. (j) Quantitative analysis of histological scores for L4‐S1 IVDs from 14‐month‐old *Smo* ^*c/c*^ and *Agc1‐CreER* ^*T2*^; *Smo* ^*c/c*^ mice. *n* = 14 discs per genotype. Data are presented as mean ± SD, with each dot representing one disc's histological score. Statistical significance was determined using the student's *t*‐test. *p < 0.05. Scale bar in panels a, b, c, g, h, i: 400 μm; 100 μm in other panels.

Collectively, our findings unravel the cellular and molecular dynamics of age‐related NP degeneration. In early stages, NPCs transdifferentiate into Krt19^+^ N‐CLCs, which subsequently progress to Krt19^−^ C‐CLCs. During advanced degeneration, these notochordal CLCs are replaced by Krt19^−^ D‐CLCs originating from non‐notochordal Gli1^+^ progenitors. While the ultimate fate of notochordal CLCs remains unresolved, they likely undergo apoptosis. Mechanistically, we demonstrate that inactivation of Hh signaling promotes the expansion of both notochordal and non‐notochordal CLC populations in mice. Further studies are required to dissect how Hh signaling inhibits CLC production. Together, these findings provide critical insights into the cellular origin of CLCs and position Hh signaling as a promising therapeutic target to counteract pathological cell fate shifts in degenerative discs.

## Author Contributions

J.C. conceived and designed the study, supervised the project, and interpreted the data. J.C. and L.Z. drafted the manuscript, incorporating input from all authors. L.Z., C.X., and H.Y. carried out the experiments and performed data analysis. All authors critically reviewed, edited, and approved the final manuscript.

## Conflicts of Interest

The authors declare no conflicts of interest.

## Supporting information


**Data S1:** acel70248‐sup‐0001‐DataS1.docx.

## Data Availability

The data that supports the findings of this study is available in the Supporting Information of this article.
