# Supplementary material for: Dual‐Lineage Chondrocyte‐Like Cells in the Nucleus Pulposus of Aging Intervertebral Discs Are Accelerated by Hedgehog Signaling Inactivation
Source: Aging Cell. 2025 Sep 22;24(11):e70248. doi: 10.1111/acel.70248 (PMC12611273; doi:10.1111/acel.70248)
Supplement: Supplementary file 1 — Data S1: acel70248‐sup‐0001‐DataS1.docx. [file ACEL-24-e70248-s001.docx]

**Supporting Information**

# 1. Materials and Methods

# 1.1 Mouse strains

Mouse strains, including *Shh-Cre* (Harfe et al., 2004), *Gli1creER^T2^* (Ahn & Joyner, 2004), *Agc1-CreER^T2^* (Henry et al., 2009)*,* *R26-mT/mG* (Muzumdar, Tasic, Miyamichi, Li, & Luo, 2007), *R26-tdTomato* (Madisen et al., 2010)*,* and *Smo^c/c^* (Long, Zhang, Karp, Yang, & McMahon, 2001) mice were generated as previously reported. To investigate the transdifferentiation of notochordal lineage cells to chondrocyte-like cells (CLCs) in the aging nucleus pulposus, *Shh-Cre* mice were crossed to *R26-mT/mG* mice to generate *Shh-Cre; R26-mT/mG* offspring*.* Intervertebral discs (IVDs) were harvested from these mice at specified ages. For lineage tracing of non-notochordal cells, *Gli1-CreER^T2^* mice were crossed to *R26-tdTomato* mice to produce *Gli1-CreER^T2^; R26-tdTomato* progeny. Tamoxifen (100 mg/kg) was administered intraperitoneally to 2-month-old mice to activate Cre recombinase, followed by analysis at designated time points. To conditionally inactivate Hedgehog (Hh) signaling in IVDs, *Agc1-CreER^T2^*; *Smo^c/c^* mice were generated and injected with tamoxifen at 2 months of age. All animals were housed in a specific pathogen-free (SPF) facility with controlled temperature (22–24°C) and 12-hour light-dark cycles. Experimental protocols adhered to the National Institutes of Health (NIH) Guide for the Care and Use of Laboratory Animals, and approved by the Experimental Animal Welfare Ethics Committee of Hangzhou City University (Approval Number: #21001).

**1.2 Tamoxifen administration**

Tamoxifen (Sigma-Aldrich, T5648) was dissolved in pre-warmed corn oil (37°C; Sigma-Aldrich, C8267) to a final concentration of 10 mg/mL. The solution was aliquoted and stored in amber glass vials at 4°C to protect from light exposure. For in vivo administration, two-month-old mice underwent daily intraperitoneal (i.p.) injections of the prepared tamoxifen solution at a dosage of 100 mg/kg body weight, administered over five consecutive days. Spinal columns were collected from mice at various time points ranging from 6 to 22 months of age.

## **Hematoxylin and Eosin (H&E) staining**

Spinal columns from euthanized mice were fixed in 4% paraformaldehyde (PFA) at 4°C for 24 hours, followed by decalcification in 14% EDTA (4°C, 14 days). Tissues were cryoprotected in 15% and then 30% sucrose (4°C, 24 hours each) before embedding in OCT compound. Sections (7 μm thickness) were stained with 2% hematoxylin (Sigma, H9627) for 1 minute, rinsed in distilled water, and counterstained with eosin for 3 minutes. After staining, samples were dehydrated through graded ethanol (75% and 100%, 1 minute each), cleared in xylene, and finally mounted with neutral resin.

## **Safranin O/Fast Green staining**

Sections were stained with hematoxylin (2 minutes), and then differentiated in 1% acid alcohol (1% HCl in 75% ethanol) and rinsed with distilled water. Counterstaining was performed with 0.02% fast green (Sigma, F7252; 2 minutes), followed by differentiation in 1% acetic acid in 75% ethanol. Sections were then stained with 1% safranin O (Sigma, S2255; 15 minutes), dehydrated in ethanol (95% and 100%), cleared in xylene, and permanently mounted with neutral resin.

## **Immunofluorescence staining**

For Krt19 staining, coronal sections underwent antigen retrieval in 10 mM Tris-EDTA (pH 9.0; 65°C, 1 hour). For ColX staining, sections were pretreated with 2 mg/mL hyaluronidase (Sigma, H3506; 55°C, 2 hours). After blocking with 10% normal goat serum (YEASEN, 36119ES; 1 hour), primary antibodies against Krt19 (Abcam, ab52625; 1:200) or ColX (Abcam, ab58632; 1:200)] were applied overnight at 4°C. The next day, sections were incubated with Alexa Fluor 647 anti-rabbit secondary antibody (Invitrogen, A21246; 1:200; 1 hour), counterstained with DAPI, and then mounted with Mowiol mounting solution.

## **Krt19 immunohistochemical staining**

Frozen sections were first baked at 55°C for 1 hour to enhance adhesion to microscope slides. Antigen retrieval was then performed by incubating the sections in 10 mM Tris-EDTA buffer (pH 9.0) at 65°C for 1 hour. Following antigen retrieval, endogenous peroxidase activity was blocked using the peroxidase blocker from a commercial IHC kit (ZSGB-BIO, PV9001) for 15 minutes at room temperature. The primary antibody against Krt19 (Abcam, ab52625; 1:200) was applied to the sections, which were then incubated overnight at 4°C. On the following day, the slides were incubated with Reaction Enhancer Solution for 1 hour to optimize the detection process, followed by treatment with Enhanced Enzyme-labeled goat Anti-rabbit IgG polymer Solution for 15 minutes at room temperature to further enhance signal detection. For signal development, DAB chromogen was applied to the sections for 2–5 minutes, resulting in brown deposits at sites of positive antigen detection. The reaction was terminated by rinsing the slides with distilled water. To provide nuclear contrast, the sections were counterstained with hematoxylin for 30 seconds. Finally, the sections were dehydrated through 75% and 100% ethanol, cleared in xylene, and then mounted with neutral resin for microscopic examination and analysis.

**1.7 Histological score assessment**

To evaluate the impact of aging or *Smo* ablation on disc degeneration, coronal IVD sections were stained with hematoxylin and eosin (H&E) or Safranin O/Fast Green. Pathological scores for the annulus fibrosus (AF) and nucleus pulposus (NP) were determined using a validated scoring system adapted for mouse IVDs (Tam et al., 2018). Individual AF and NP scores were summed to generate a total histological score per disc.

**1.8 Statistics**

Statistical significance was determined using Student’s t-test to compare histological scores between Smo^c/c^ and *Agc1-CreER^T2^; Smo^c/c^* mice. For histological scores across different categories of intervertebral discs in aging *Shh-Cre; mT/mG* mice, one-way analysis of variance (ANOVA) with Tukey’s multiple comparisons test was applied. A threshold of *p < 0.05* was considered statistically significant. All analyses were performed using GraphPad Prism 8 (GraphPad Software, San Diego, CA). Data are presented as mean ± SD, with each dot representing individual disc data.

**References**

Ahn, S., & Joyner, A. L. (2004). Dynamic changes in the response of cells to positive hedgehog signaling during mouse limb patterning. *Cell, 118*(4), 505-516. doi:10.1016/j.cell.2004.07.023

Harfe, B. D., Scherz, P. J., Nissim, S., Tian, H., McMahon, A. P., & Tabin, C. J. (2004). Evidence for an expansion-based temporal Shh gradient in specifying vertebrate digit identities. *Cell, 118*(4), 517-528. doi:10.1016/j.cell.2004.07.024

Henry, S. P., Jang, C. W., Deng, J. M., Zhang, Z., Behringer, R. R., & de Crombrugghe, B. (2009). Generation of aggrecan-CreERT2 knockin mice for inducible Cre activity in adult cartilage. *Genesis, 47*(12), 805-814. doi:10.1002/dvg.20564

Long, F., Zhang, X. M., Karp, S., Yang, Y., & McMahon, A. P. (2001). Genetic manipulation of hedgehog signaling in the endochondral skeleton reveals a direct role in the regulation of chondrocyte proliferation. *Development, 128*(24), 5099-5108. doi:10.1242/dev.128.24.5099

Madisen, L., Zwingman, T. A., Sunkin, S. M., Oh, S. W., Zariwala, H. A., Gu, H., . . . Zeng, H. (2010). A robust and high-throughput Cre reporting and characterization system for the whole mouse brain. *Nat Neurosci, 13*(1), 133-140. doi:10.1038/nn.2467

Muzumdar, M. D., Tasic, B., Miyamichi, K., Li, L., & Luo, L. (2007). A global double-fluorescent Cre reporter mouse. *Genesis, 45*(9), 593-605. doi:10.1002/dvg.20335

Tam, V., Chan, W. C. W., Leung, V. Y. L., Cheah, K. S. E., Cheung, K. M. C., Sakai, D., . . . Chan, D. (2018). Histological and reference system for the analysis of mouse intervertebral disc. *J Orthop Res, 36*(1), 233-243. doi:10.1002/jor.23637

**Supplementary Figures**

**
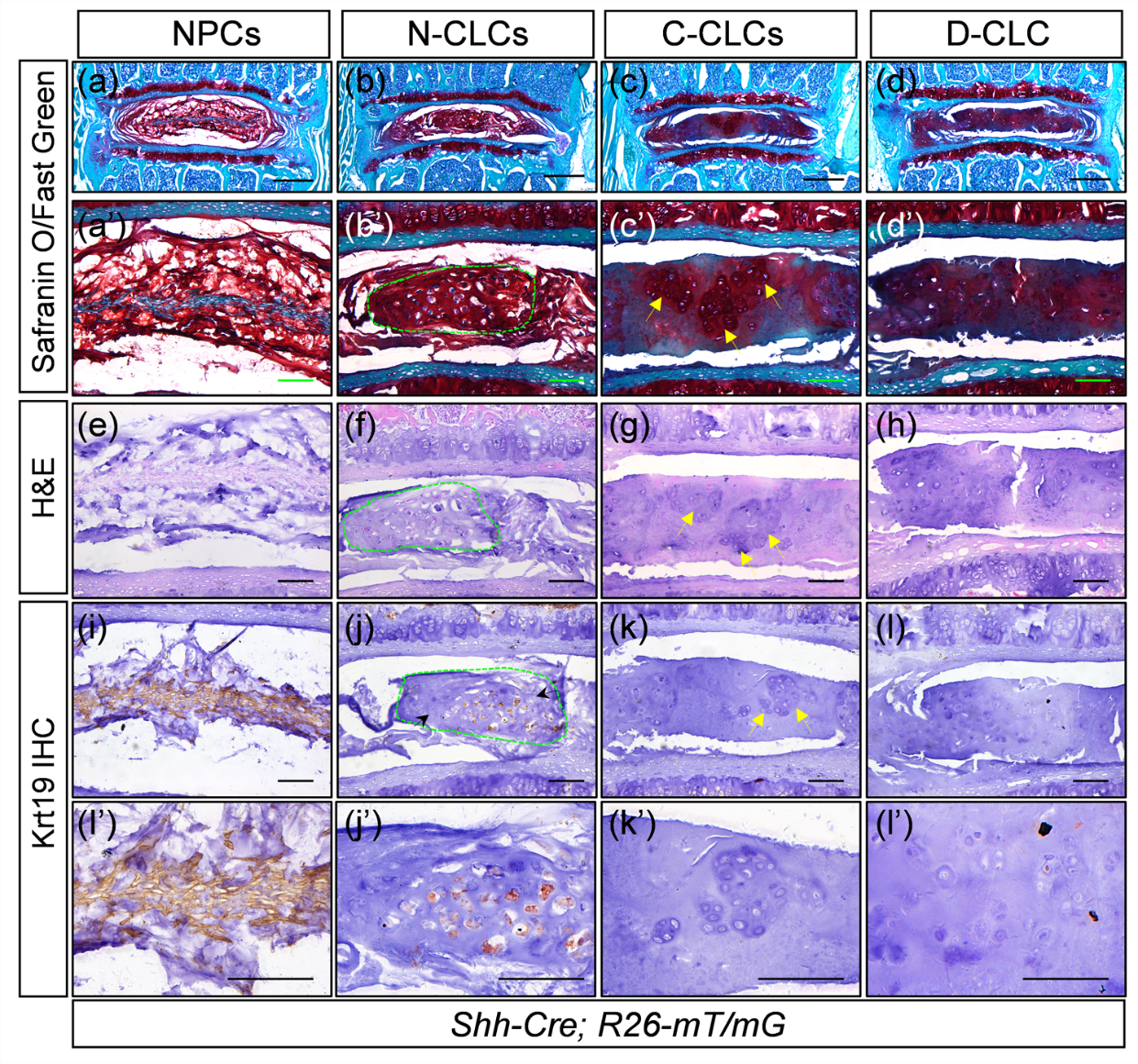
**

**Supplementary Figure 1.** Evaluation of intervertebral disc (IVD) pathology of 16- to 24-month-old *Shh-Cre; R26-mT/mG* mice via histological and Krt19 immunohistochemical (IHC) analysis. **(a–l)** Safranin O/Fast Green (a–d’), hematoxylin and eosin (H&E) (e–h), and Krt19 IHC staining (i–l) of the coronal IVD sections of from 16- to 24-month-old *Shh-Cre; mT/mG* mice. Panels a, b, c, and d show low-power views of entire intervertebral discs, while panels a’–d’, e–h, and i–l are high-power views of the nucleus pulposus region. Panels i'–l' show enlarged views of panels i–l, highlighting the nucleus pulposus regions containing NPC, N-CLC, C-CLC, and D-CLC, respectively. Green dashed lines in panels b’, f, j highlight nested chondrocyte-like cells (N-CLCs). Black arrows in panel j indicate N-CLCs lacking Krt19 expression. Yellow arrows in panels c’, g, k point to clustered chondrocyte-like cells (C-CLCs). Scale bar in panels a, b, c, d: 400 μm; 100 μm in other panels.


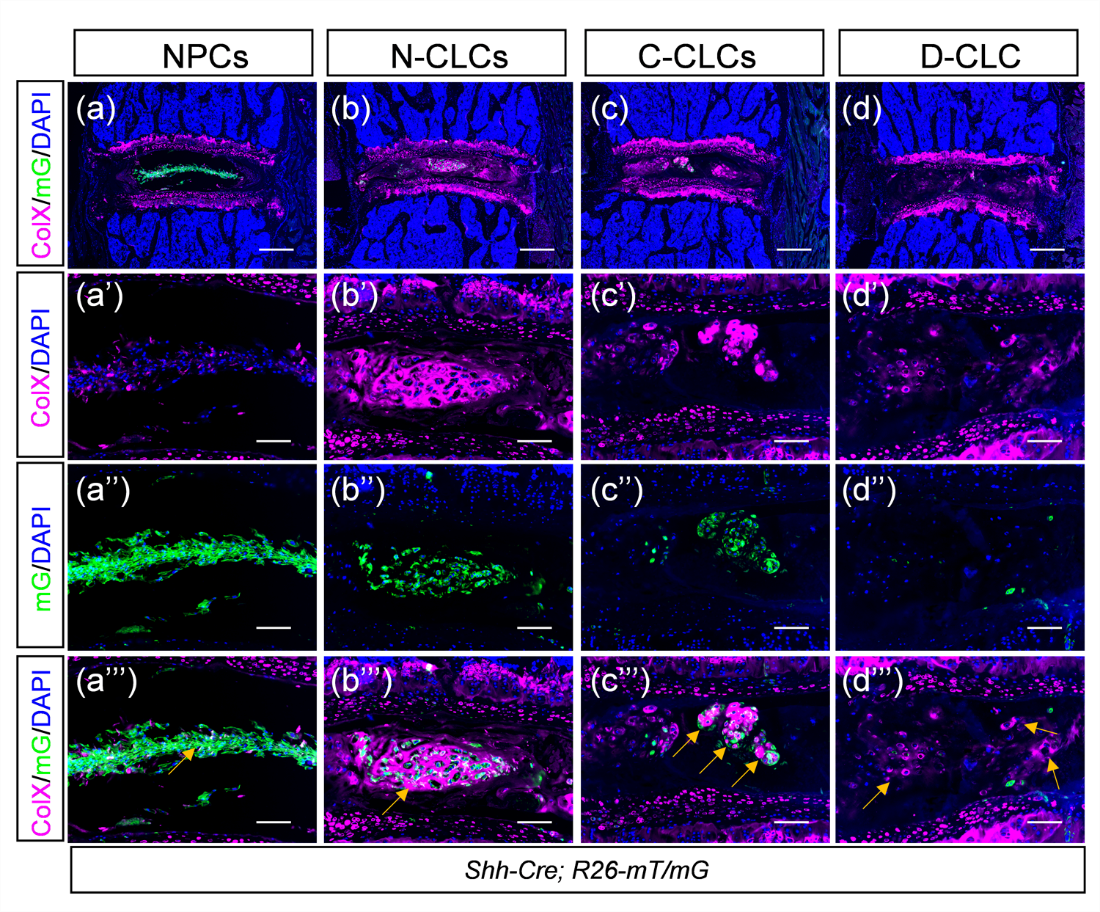


**Supplementary Figure 2.** Immunofluorescence analysis of ColX protein expression in nucleus pulposus cells (NPCs) and chondrocyte-like cells (CLCs) of intervertebral discs (IVDs) from aging *Shh-Cre; R26-mT/mG* mice. ColX and EGFP co-localization was assessed in NPCs, nested chondrocyte-like cells (N-CLCs), clustered chondrocyte-like cells (C-CLCs), and disordered chondrocyte-like cells (D-CLCs) of 16- to 24-month-old *Shh-Cre; mT/mG* mice. **(a–d)** Low-power images showing combined visualization of mG epifluorescence (green), DAPI (blue), and ColX (magenta) immunofluorescence across entire IVDs. High-power magnifications of the nucleus pulposus (NP) region are shown in (a'–a'''), (b'–b'''), (c'–c'''), and (d'–d'''), respectively. **(a'–d')** DAPI (blue) and ColX (magenta). **(a''–d'')** DAPI (blue) and mG (green). **(a'''–d''')** Combined visualization of mG (green), DAPI (blue), and ColX (magenta). Scale bar in panels a, b, c, d: 400 μm; 100 μm in other panels. Yellow arrows in **(a'''–d''')** highlight NPCs, N-CLCs, C-CLCs, and D-CLCs, respectively. Notably, ColX expression is predominantly observed in CLCs (N-CLCs, C-CLCs, D-CLCs), with minimal or absent staining in NPCs. Co-localization of ColX and EGFP (mG) is evident in CLCs.


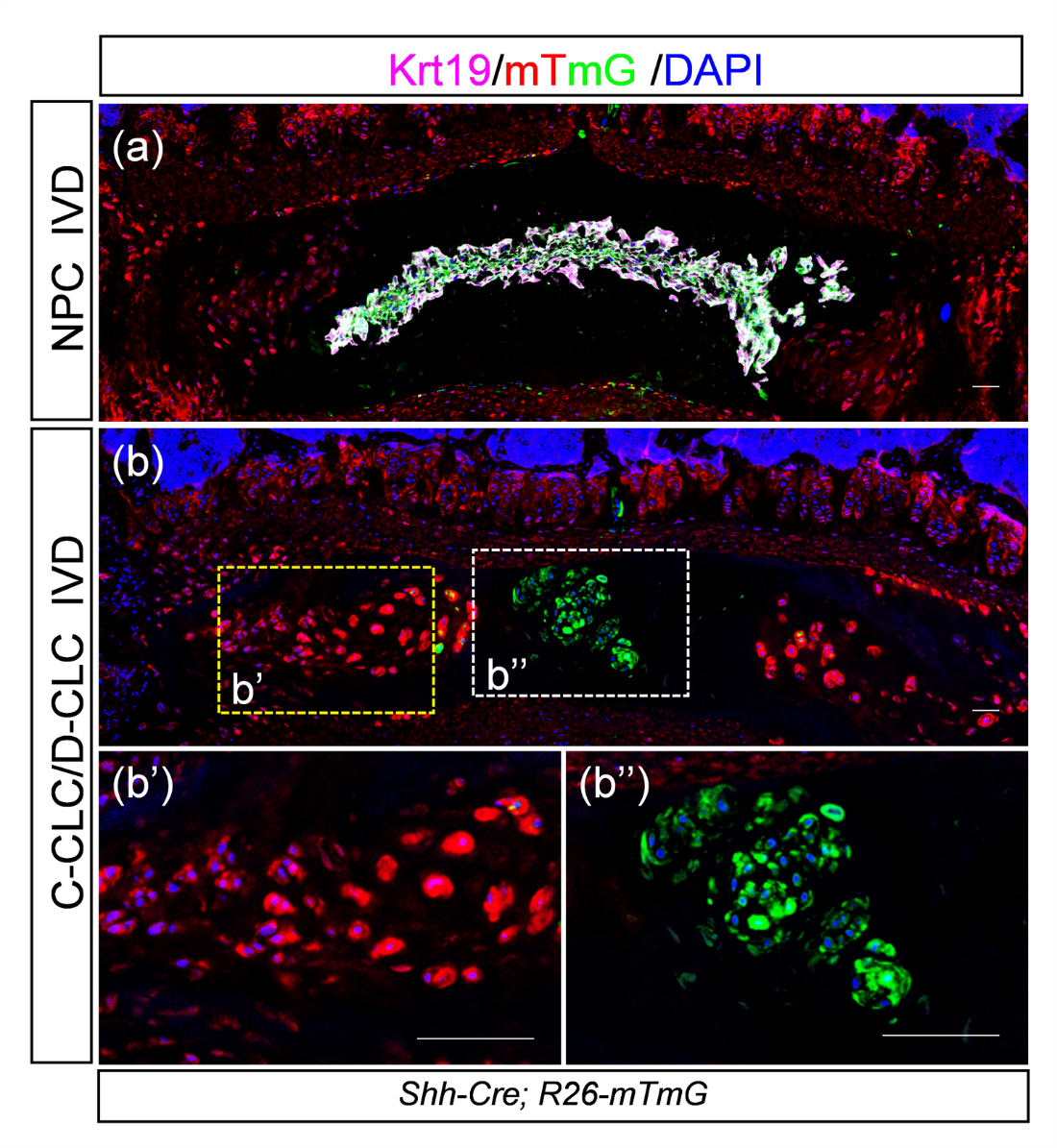


**Supplementary Figure 3.** Immunofluorescence analysis of Krt19 protein expression in nucleus pulposus cells (NPCs) and chondrocyte-like cells (CLCs) of intervertebral discs (IVDs) from aging *Shh-Cre; R26-mT/mG* mice. Krt19 and EGFP co-localization was assessed to identify NPCs and different types of CLCs in *Shh-Cre; mT/mG* mice. (a–b) Low-power combined visualization of mT (red), mG (green), DAPI (blue), and Krt19 (magenta) immunofluorescence across entire IVDs. High-power views of the encircled NP regions in (b) highlight clustered chondrocyte-like cells (C-CLCs) (b’’) and disordered chondrocyte-like cells (D-CLCs) (b’). Scale bar: 50 μm.


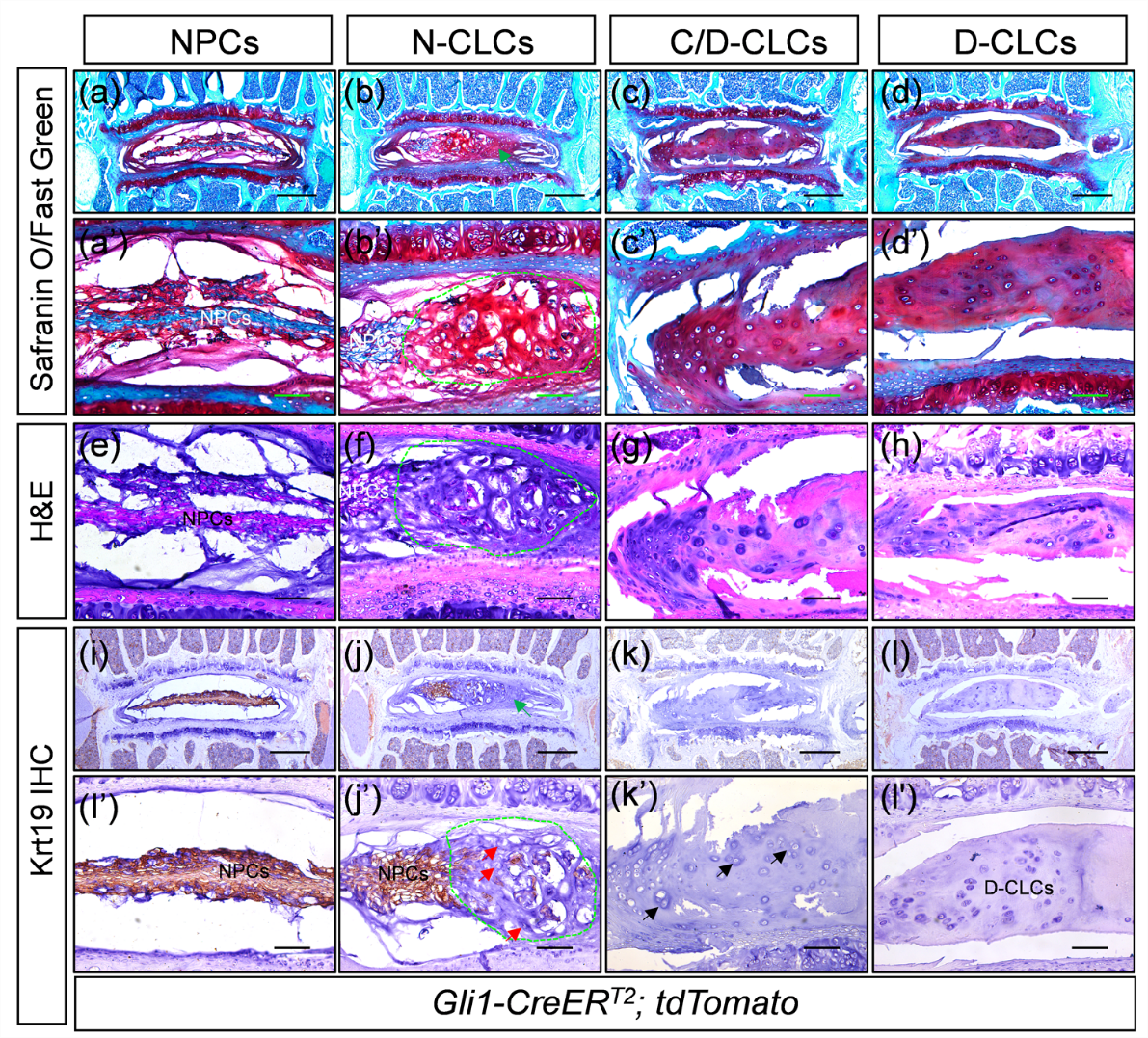


**Supplementary Figure 4.** Evaluation of intervertebral disc (IVD) pathology of aging *Gli1-CreER^T2^; tdTomato* mice via histological and Krt19 immunohistochemical (IHC) analysis. **(a–d’)** Safranin O/Fast Green staining of coronal IVD sections from 16- and 19-month-old *Gli1-CreER^T2^; tdTomato* mice. (**e–h**) Hematoxylin and eosin (H&E) staining of coronal IVD sections from 16- and 19-month-old *Gli1-CreER^T2^; tdTomato* mice. (**i–l’**) Krt19 IHC staining of coronal IVD sections. Panels a, b, c, d, i, j, k, l show low-power views of entire intervertebral discs, and corresponding high-power views of the nucleus pulposus region are shown in panels a’, b’, c’, d’, i’, j’, k’, l’, respectively. Green dashed lines in panels b’, f, j’ highlight nested chondrocyte-like cells (N-CLCs). Green arrows in panel b and j indicate the NP regions containing D-CLCs. Red arrows in panel j’ indicate N-CLCs lacking Krt19 expression. Black arrows in panel k’ point to clustered chondrocyte-like cells (C-CLCs). NPCs: nucleus pulposus cells; D-CLCs: disordered chondrocyte-like cells. Scale bar in panels a, b, c, d, i, j, k, l: 400 μm; 100 μm in other panels.


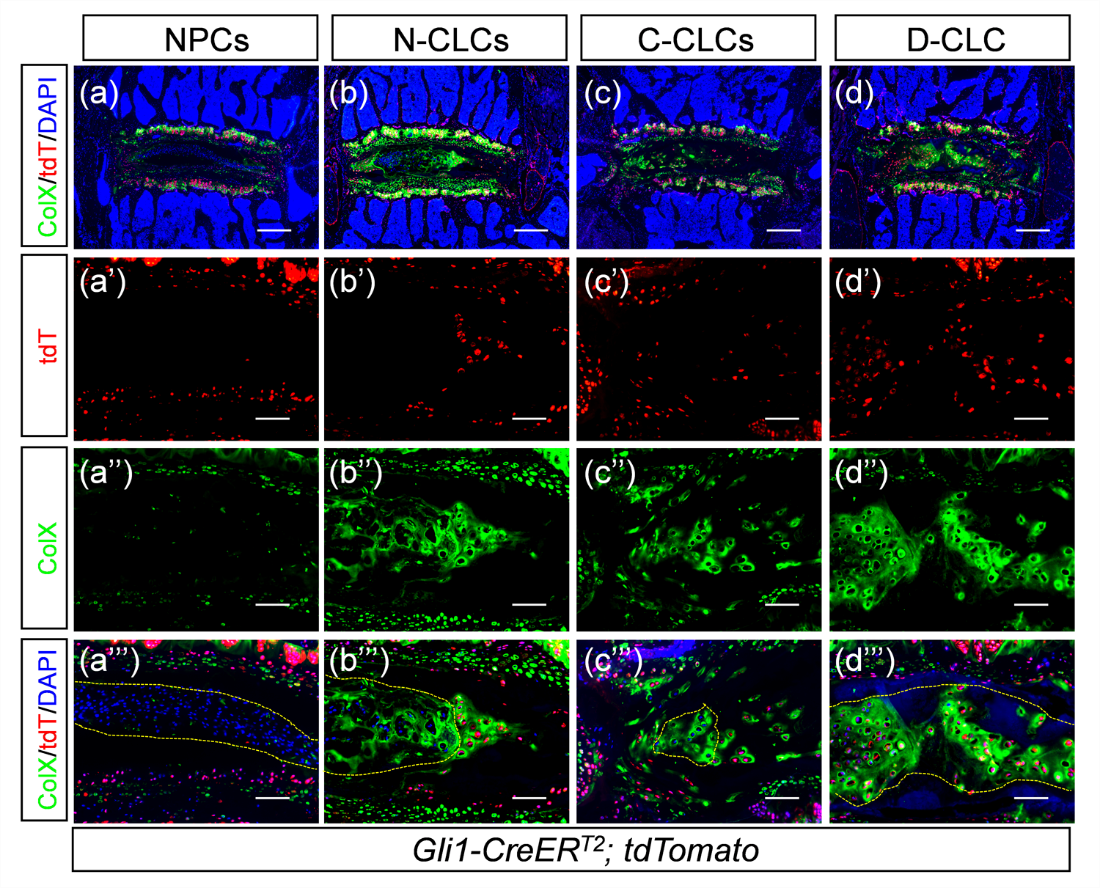


**Supplementary Figure 5.** Immunofluorescence analysis of ColX protein expression in nucleus pulposus cells (NPCs) and chondrocyte-like cells (CLCs) of intervertebral discs (IVDs) from aging *Gli1-CreER^T2^; tdTomato* mice. ColX and tdTomato (tdT) co-localization was assessed in NPCs, nested chondrocyte-like cells (N-CLCs), clustered chondrocyte-like cells (C-CLCs), and disordered chondrocyte-like cells (D-CLCs) of 16- and 19-month-old *Gli1-CreER^T2^; tdTomato* mice. **(a–d)** Low-power images of entire IVDs showing combined visualization of tdT epifluorescence (red), DAPI (blue), and ColX (green) immunofluorescence. High-power magnifications of the nucleus pulposus (NP) region are shown in (a'–a'''), (b'–b'''), (c'–c'''), and (d'–d'''), respectively. **(a'–d')** tdT epifluorescence. **(a''–d'')** ColX immunofluorescence. **(a'''–d''')** Combined visualization of tdT (red), DAPI (blue), and ColX (green). Scale bar in panels a, b, c, d: 400 μm; 100 μm in other panels. The encircled NP regions in panels a''', b''', c''', and d''' indicate nucleus pulposus cells (NPCs), nested chondrocyte-like cells (N-CLCs), clustered chondrocyte-like cells (C-CLCs), and disordered chondrocyte-like cells (D-CLCs), respectively. Notably, ColX expression is predominantly observed in CLCs (N-CLCs, C-CLCs, D-CLCs) but is minimal or absent in NPCs. Co-localization of tdT and ColX is exclusively detected in D-CLCs.

**
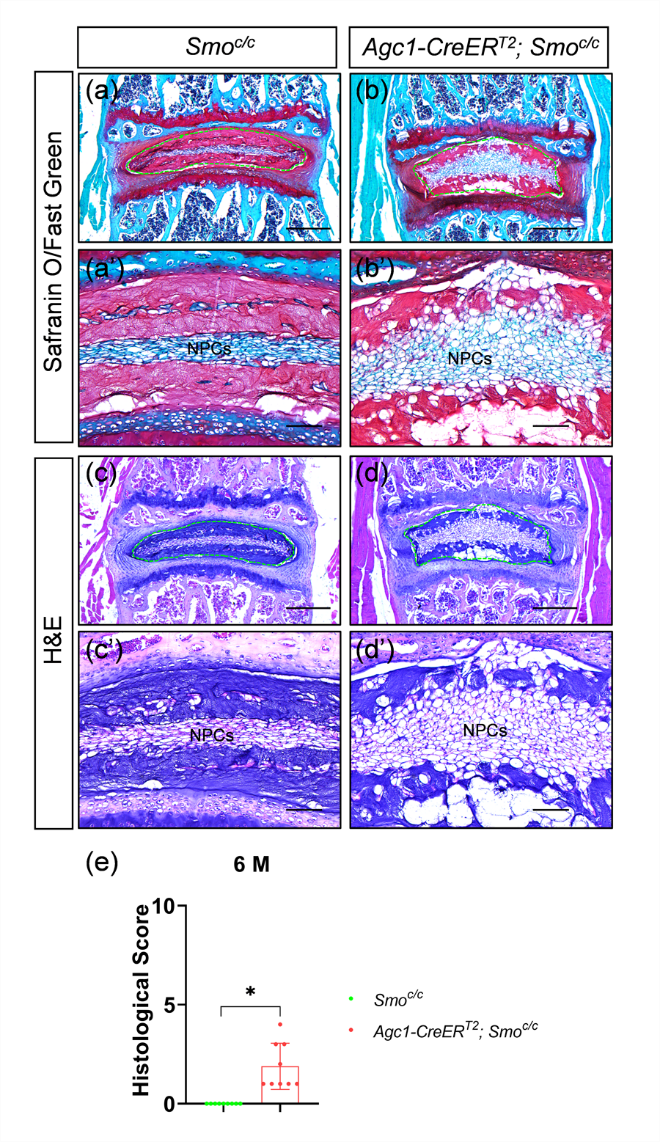
**

**Supplementary Figure 6.** Histological analyses of lower lumbar intervertebral discs from 6-month-old *Smo^c/c^* and *Agc1-CreER^T2^; Smo^c/c^* mice. **(a–b’)** Safranin O/Fast Green staining of coronal sections from 6-month-old *Smo^c/c^* (a, a’) and *Agc1-CreER^T2^; Smo^c/c^* mice (b, b’). **(c–d’)** Hematoxylin and eosin (H&E) staining of coronal sections from 6-month-old *Smo^c/c^* (c, c’) and *Agc1-CreER^T2^; Smo^c/c^* mice (d, d’). Panels a, b, c, d show low-power views of entire intervertebral discs, with the nucleus pulposus region encircled by a green dashed line. Panels a’, b’, c’, d’ are high-power views of the nucleus pulposus region corresponding to panels a, b, c, d, respectively. NPCs: nucleus pulposus cells. Scale bar in panels a, b, c, d: 400 μm; 100 μm in other panels. **(e)** Quantitative analysis of histological scores for lower lumbar discs (L4-L5, L5-L6, L6-S1) from 6-month-old *Smo^c/c^* and *Agc1-CreER^T2^; Smo^c/c^* mice. n=9 discs per genotype.
